# CAZyme fold architecture is conserved between disparate environments despite extreme sequence divergence

**DOI:** 10.1128/msystems.00485-26

**Published:** 2026-05-19

**Authors:** Oliyad Jeilu, Addis Simachew, Erica M. Hartmann, Erik Alexandersson, Eva Johansson

**Affiliations:** 1 Department of Plant Breeding, Swedish University of Agricultural Sciences 8095, Lomma, Sweden; 2 Department of Civil and Environmental Engineering, Northwestern University 3270, Evanston, Illinois, USA; 3 Center for Synthetic Biology, Northwestern University 3270, Evanston, Illinois, USA; 4 Biotechnology Research Centre, Institute of Advanced Science and Technology, Addis Ababa University 37602, Addis Ababa, Ethiopia; 5 Department of Medicine, Division of Pulmonary and Critical Care, Northwestern University 205058, Chicago, Illinois, USA; Universidad Miguel Hernandez de Elche, San Juan de Alicante, Alicante, Spain

**Keywords:** shotgun metagenomics, soda lakes, rumen microbiome, biogeochemical cycles, carbon cycling, CAZymes, MAGs, microbial diversity

## Abstract

Microbial carbohydrate-active enzymes (CAZymes) underpin carbon cycling across Earth’s ecosystems; however, how contrasting environments shape CAZyme diversity and structural conservation remains poorly understood. Here, we applied shotgun metagenomics to compare the carbohydrate-degradation potential of two functionally prolific but physicochemically opposed ecosystems: the alkaline-saline soda lakes of the East African Rift Valley and the anaerobic ruminant gut. From 34 metagenomes (12 soda lake and 22 rumen), we recovered 371 quality-filtered metagenome-assembled genomes, of which 84% of soda lake and 52% of rumen MAGs represented novel species. Rumen communities, dominated by *Bacteroidota*, *Fibrobacterota*, and *Bacillota*, exhibited significantly higher taxonomic diversity and were enriched in carbohydrate catabolism and fermentation pathways. Soda lake communities, dominated by *Pseudomonadota*, displayed greater evolutionary divergence (lower RED scores) and were enriched in pH homeostasis, oxidative and osmotic stress, sulfur cycling, and carbon fixation pathways. To assess whether structural conservation persists despite extreme sequence divergence, we predicted three-dimensional structures for 12 representative enzymes from six glycoside hydrolase families (GH1, GH3, GH5_11, GH9, GH10, and GH28) using AlphaFold 3. All 12 structures adopted canonical GH family folds with high confidence (pTM 0.75–0.97). These results demonstrate that environmental selection drives distinct taxonomic and functional strategies for carbon processing while preserving three-dimensional CAZyme architecture, positioning soda lake and rumen metagenomes as complementary reservoirs for bioprospecting industrially relevant enzymes.

**IMPORTANCE:**

Carbohydrate-active enzymes, or CAZymes, are the molecular machines that microorganisms use to break down plant material and other complex sugars, and they underpin both the global carbon cycle and many industrial processes, from biofuel production to food, feed, and textile manufacturing. In this study, we compared the CAZyme repertoires of two microbial worlds that could hardly be more different: the alkaline, salty soda lakes of the East African Rift Valley, and the anaerobic stomachs of cattle, sheep, and goats. We show that although these communities are taxonomically distinct and their enzyme sequences have diverged dramatically, the three-dimensional shapes of their key carbohydrate-degrading enzymes remain remarkably well preserved. Soda lakes, in particular, hold a large pool of previously uncharacterised enzymes, identifying them as a promising, largely untapped source of robust biocatalysts for sustainable biotechnology and industrial applications.

## INTRODUCTION

Microbial life thrives in diverse environments, from chemically harsh ecosystems to highly specialized biological niches. Microbial metabolism is central to carbon cycling. Beyond the primary productivity of carbon fixation, this process is fundamentally driven by enzyme-mediated depolymerization of polysaccharides, positioning carbohydrate-active enzymes (CAZymes) as central determinants of ecosystem function. Many ecosystems host dense and metabolically active microbial consortia, specialized in enzymatic transformations related to carbon cycling, fiber degradation, and energy production (1–5). This shared reliance on CAZyme-mediated carbon processing, despite contrasting environmental pressures, creates a powerful natural experiment to compare whether similar metabolic demands produce convergent enzymatic solutions or different selection pressures drive fundamentally different evolutionary strategies.

Soda lakes are unique poly-extreme environments characterized by high alkalinity (typically pH 9–11) and elevated salinity, offering a distinctive ecological niche for diverse microbial communities (6). These environments, found globally but especially abundant along the East African Rift Valley, harbor microorganisms capable of surviving and thriving under extreme conditions (7, 8). Extremophilic microbes in these environments also play essential, often uncharacterized, roles in global biogeochemical cycling. Ethiopian soda lakes, including Lakes Abijata, Chitu, and Shala, are renowned for their high primary productivity and diverse microbial communities, encompassing bacteria, archaea, and eukaryotes (7, 9, 10).

Parallel to these extreme but productive soda lake ecosystems, another biologically dense and metabolically dynamic microbial habitat exists: the rumen of ruminants. The rumen is a specialized digestive organ in ruminants like cattle, goats, and sheep, where microbial fermentation breaks down plant biomass into usable nutrients for the host animal (11). This fermentation process is not only vital for host nutrition but is also a critical component of the global carbon cycle, as methanogenic archaea in the rumen are a primary source of anthropogenic greenhouse gas emissions. The rumen is a warm (~39°C–40°C), slightly acidic (pH ~5.5–6.8), and anaerobic environment. The rumen microbiome is one of the most complex among the wide array of microbial ecosystems present on Earth (11, 12) and represents the largest characterized metagenomic source of carbohydrate-active enzymes (13). Because of this unparalleled efficiency in the degradation of complex plant polymers, we believe that the rumen microbiome can be regarded as a benchmark system for natural biomass conversion.

Despite their environmental differences, soda lakes and rumen face a similar biochemical challenge: the enzymatic degradation of complex carbohydrates; however, they do so under radically different physicochemical regimes. Comparing these systems, therefore, provides a powerful framework to evaluate whether similar metabolic outcomes are achieved through convergent enzyme repertoires or through fundamentally different evolutionary strategies. In soda lakes, microbial communities rely on dissolved inorganic carbon, primarily bicarbonate and carbonate, as the basis for primary production. Photosynthetic microbes, particularly cyanobacteria, convert these compounds into organic biomass, while heterotrophic alkaliphiles metabolize dissolved organic carbon derived from decaying microbial mats and extracellular polymers (7, 14, 15). In contrast, the rumen microbiome specializes in the anaerobic fermentation of plant-derived polysaccharides, including cellulose, hemicellulose, lignin, and starch. These polymers are broken down into volatile fatty acids (VFAs) such as acetate, propionate, and butyrate, which serve as the primary energy source for the host animal. The rumen microbiome’s enzymatic repertoire includes fibrolytic enzymes adapted to low-oxygen conditions, functioning through syntrophic interactions among bacteria, protozoa, and methanogens (5, 16).

Building on our previous functional metagenomic discovery of novel CAZymes from these soda lakes and characterization of an alkaline-adapted GH3 β-glucosidase (4, 17, 18), we now apply shotgun metagenomics to move beyond individual enzyme discovery toward ecosystem-level comparison. While functional metagenomics excels at identifying specific enzymes, it lacks the capacity to comprehensively characterize microbial community structure and ecosystem-level functionality (19–21). Shotgun metagenomics overcomes these limitations by enabling simultaneous reconstruction of microbial lineages, metabolic pathways, and enzyme repertoires, while also permitting genome-resolved analyses of evolutionary features such as genome architecture and phylogenetic divergence (19, 22). We hypothesize that convergent metabolic demands are met through divergent evolutionary strategies: specifically, that soda lake communities achieve carbohydrate processing through adapting the extreme environment and evolve novel but structurally conserved CAZymes carried by a lower diversity of functionally versatile generalist lineages. However, the rumen achieves equivalent function through a higher diversity of specialist taxa deploying a more conserved enzymatic toolkit. To test this, we integrate metagenomics with CAZyme profiling, AlphaFold3 structure prediction, and Foldseek structural similarity analysis across samples from Ethiopian soda lakes and the ruminant microbiome.

## MATERIALS AND METHODS

### Sampling sites and collection procedures

Sediment samples were collected from three East African Rift Valley soda lakes: Lakes Abijata, Chitu, and Shala, as described in Jeilu et al. (18). Rumen content samples were aseptically collected from freshly slaughtered goats, cattle, and sheep at the Addis Ababa Abattoirs Enterprise. Immediately post-mortem, approximately 50 mL of rumen content was extracted from each animal using sterile instruments and transferred into sterile 50 mL Falcon tubes. Samples were immediately placed on ice and transported to the laboratory under cold chain conditions. All samples were processed within 24 h of collection to preserve microbial integrity. The pH of all samples was measured using a pH meter (OAKTON-pH110), and the salinity of soda lake samples was measured using a refractometer (HHTEC). Soda lake samples are identified by lake-specific prefixes: ABS/AS/ASP (Abijata), CHS/CW/CSP/CLP (Chitu), and SHL/SWL/SLP (Shala). Rumen samples are prefixed by host species initial (G = goat, C = cattle, and S = sheep).

### DNA extraction

DNA from soda lake samples was extracted using the cetyltrimethylammonium bromide (CTAB)/sodium dodecyl sulfate (SDS) method, as described in a previous study (18). This protocol was optimized for high-yield and high-purity DNA recovery from environmental samples collected from soda lakes. DNA extraction from rumen samples (goat, cattle, and sheep) was performed using the DNeasy PowerSoil Kit (QIAGEN), following the manufacturer’s instructions. Briefly, 0.25 g of each sample was transferred into a PowerBead Tube containing lysis buffer and subjected to bead beating using a vortex adapter. The supernatant was separated by centrifugation, and inhibitors were removed using Inhibitor Removal Technology (IRT) reagents. DNA was then bound to a silica membrane within a spin column, washed to remove contaminants, and eluted in 50 μL of TE buffer (pH 8.0). DNA quality and concentration were assessed using a NanoDrop spectrophotometer (Thermo Scientific) and agarose gel electrophoresis.

### Shotgun metagenomic sequencing

Shotgun metagenomic sequencing was performed by Novogene using the Illumina platform (PE150 reads, paired-end 150 bp). Libraries were prepared according to Novogene’s metagenomics library preparation protocol to ensure high-quality sequencing output. Each sample was sequenced to a minimum depth of 5 gigabases (Gb). Quality control assessments ensured that the proportion of bases with a Q30 score (≥85%) was maintained to guarantee high sequencing accuracy. Raw sequencing reads underwent standard quality filtering, adapter trimming, and preprocessing before downstream bioinformatics analyses, as described below.

### Bioinformatics analysis

Raw sequencing reads were assessed using FastQC (23) to evaluate sequence quality. Adapters and low-quality bases were trimmed using Trimmomatic (24) with the parameters SLIDINGWINDOW:4:20 MINLEN:50. Contaminant sequences, including human DNA and PhiX control DNA, were filtered using KneadData (https://huttenhower.sph.harvard.edu/kneaddata/) under default parameters. Overrepresented sequences were identified and removed. A final quality check was conducted using FastQC**,** and summary reports were compiled using MultiQC (25). Sequencing coverage effort analysis was performed using Nonpareil v3.5.5 (26) to estimate microbial diversity and evaluate sequencing depth across samples.

Taxonomic profiling of metagenomic reads was conducted using MetaPhlAn4 (27), employing the Jun23_CHOCOPhlAnSGB_202307 database. The resulting taxonomic profiles were analyzed to assess microbial community composition and diversity indices.

The metagenomic reads were assembled into contigs using MEGAHIT v1.2.9 (28) with default parameters. Assembly quality was assessed using QUAST v5.2.0 (29). MAGs were reconstructed using MetaBAT 2 (30) by aligning reads to the assembled contigs using Bowtie2 v2.5.4 (31). BAM files were sorted and indexed using Samtools v1.20 (32). The completeness and contamination of the assembled MAGs were assessed using CheckM v1.1.6 (33). MAGs were classified as high quality (completeness 90% or above, contamination 5% or below), medium quality (completeness 50% or above, contamination 10% or below), or low quality (below these thresholds). To remove redundancy from closely related MAGs recovered across multiple samples, the medium-to-high-quality MAGs were dereplicated using dRep v3.4.2 (34) at 95% average nucleotide identity (ANI), consistent with the species-level threshold. Only MAGs with ≥50% completeness and ≤10% contamination were retained for downstream analyses.

Taxonomic classification of MAGs was performed using GTDB-Tk v2.4.0 (35), with the most recent GTDB Release 220. In addition to taxonomic assignment, relative evolutionary divergence (RED) values were obtained from GTDB-Tk outputs and used to assess the evolutionary novelty of the reconstructed MAGs. A phylogenomic tree of MAGs was constructed using PhyloPhlAn v3.0 (36) with the GTDB-Tk reference tree as a backbone. GTDB-Tk places query genomes onto the pre-computed bacterial and archaeal reference tree using pplacer after identifying marker genes with HMMER; the resulting tree was then refined through PhyloPhlAn (36) and pruned to retain only the query MAGs.

Functional annotation was conducted using Prokka v1.14.5 (37) to identify coding sequences, rRNAs, and tRNAs. Carbohydrate-active enzymes (CAZymes) (38) were identified using dbCAN3, which integrates HMMER, DIAMOND, and dbCAN_sub for CAZyme classification.

### Selection of CAZyme candidates for structural analysis

Six GH families with direct relevance to lignocellulose bioconversion were selected for structural prediction: GH1 and GH3 (β-glucosidases, rate-limiting saccharification step), GH5 subfamily 11 and GH9 (endoglucanases, cellulose backbone cleavage), GH10 (endo-xylanase, hemicellulose hydrolysis), and GH28 (polygalacturonase, pectin degradation). All six families were represented in both soda lake and rumen environments. For each family, the top-ranked candidate sequence from each environment was extracted from the full dbCAN-annotated CAZyme catalog, prioritizing the highest DIAMOND percent identity to a characterized CAZy reference and the strongest dbCAN annotation consensus (number of supporting tools: HMMER, DIAMOND, and dbCAN_sub).

### Protein structure prediction

Three-dimensional protein structures were predicted for all 12 candidate sequences using the AlphaFold Server (https://alphafoldserver.com), which implements AlphaFold3 (39), with full genetic database multiple sequence alignment generation. All predictions were run using default server parameters with five model replicates per sequence. Prediction confidence was assessed using the predicted template modeling score (pTM), where scores >0.7 indicate high confidence in the overall fold topology, and the predicted local distance difference test (pLDDT), which estimates per-residue model accuracy on a scale of 0–100. Predicted structures were compared against the PDB100 clustered structure database using Foldseek (40, 41). Structural similarity searches were performed using the local distance difference test (lddt) alignment metric with an e-value threshold of 0.001. The best-scoring hit per query structure was identified by selecting the maximum lddt score. Structural similarity was assessed using lddt, where scores >0.7 indicate high structural similarity and scores >0.5 indicate shared fold topology (41).

### Structural superposition and active site annotation

Superposition of soda lake and rumen homologues within each GH family was performed using PyMOL (v3.1) (40, 42) to calculate root mean square deviation (RMSD) of Cα atoms. Known catalytic residues were obtained from the CAZy 3D structure-function database and mapped onto predicted structures using structural superposition against characterized reference structures. For each GH family, the canonical catalytic residues (acid/base catalyst and nucleophile) were annotated, and conservation status was recorded as conserved (identical residue at the equivalent structural position) or substituted. Structure visualization was performed in PyMOL with cartoon representation colored by per-residue pLDDT confidence (blue >90, cyan 70–90, yellow 50–70, orange <50), catalytic residues shown as red sticks, and disordered regions (pLDDT < 50) hidden to highlight the structured core.

### Statistical analysis and visualization

All downstream statistical analyses and visualizations were conducted in R v4.3.1 (43) using the vegan, phyloseq, ggplot2, ComplexHeatmap, and patchwork packages. Alpha diversity was quantified as observed species richness and Shannon diversity index (vegan package), with differences between environments assessed by Kruskal–Wallis rank-sum tests. Beta diversity was calculated using Bray–Curtis dissimilarity matrices (vegdist function, vegan), ordinated by principal coordinates analysis (PCoA; cmdscale) and non-metric multidimensional scaling (NMDS), and differences in community composition between environments were tested by permutational multivariate analysis of variance (PERMANOVA; adonis2, 999 permutations). Differences in MAG quality metrics, relative evolutionary divergence (RED) values, and average nucleotide identity (ANI) to closest GTDB reference genomes between environments were compared using Kruskal–Wallis tests. CAZyme diversity per MAG (class richness and Shannon index) and sequence identity distributions to characterized CAZy references were compared between soda lake and rumen MAGs using Kruskal–Wallis and Wilcoxon rank-sum tests, respectively. The phylogenomic tree was visualized as a circular cladogram using ggtree and treeio, with phylum-level annotations displayed as an outer ring (gheatmap). All *P*-values were considered significant at α = 0.05 unless otherwise stated.

## RESULTS

### Microbial community composition of soda lake and rumen environments

**Fig 1:**
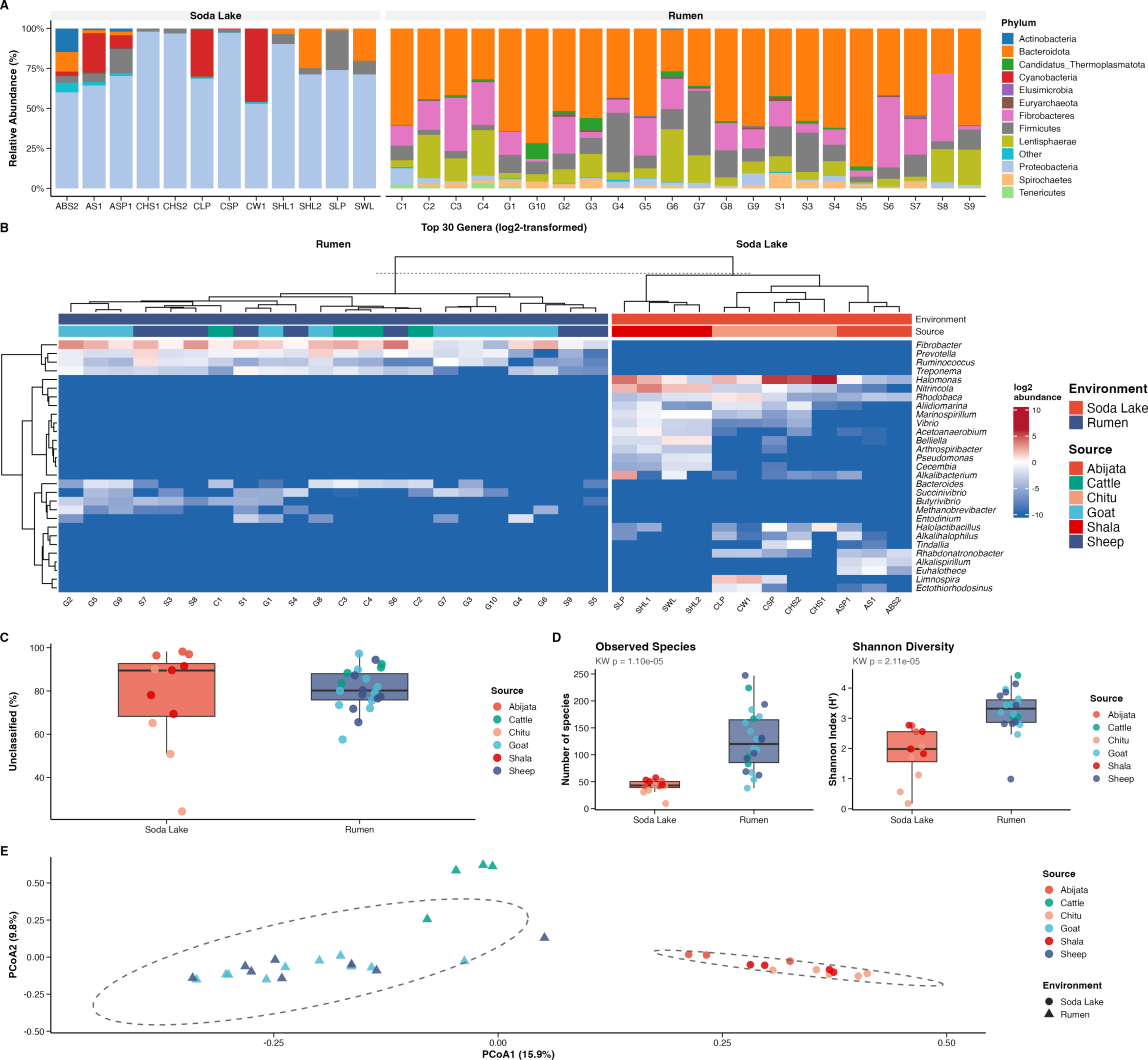
Community composition of soda lake and rumen microbiomes. (**A**) Phylum-level composition per sample. (**B**) Top 30 genera heatmap (log10 relative abundance). (**C**) Proportion of unclassified reads. (**D**) Alpha diversity (Shannon index) comparison. (**E**) PCoA ordination based on Bray–Curtis dissimilarity.

Shotgun metagenomics was performed on 34 samples from three Ethiopian soda lakes (Abijata, Chitu, and Shala; *n* = 12) and three ruminant species (goat, cattle, and sheep; *n* = 22). Taxonomic profiling using MetaPhlAn4 revealed divergent community structure between the two environments (Fig. 1A). Soda lake communities were dominated by *Proteobacteria* with lesser contributions from *Bacillota* (*Firmicutes*) and *Cyanobacteria*. In contrast, rumen communities exhibited a markedly different profile dominated by *Bacteroidota*, *Fibrobacteres*, *Bacillota* (*Firmicutes*), and *Lentisphaerae*. At the genus level, *Halomonas* overwhelmingly dominated soda lake samples, with *Nitrincola* and *Limnospira* as secondary contributors. Rumen communities were dominated by *Fibrobacter*, *Prevotella*, and *Ruminococcus* (Fig. 1B). A substantial fraction of reads remained unclassified in both environments (soda lake median: 89.5%; rumen median: 80.2%; Fig. 1C). In addition, rumen samples exhibited significantly higher species-level alpha diversity than soda lake samples (Kruskal–Wallis *P* < 0.001 for both observed species and Shannon index; Fig. 1D). Beta-diversity analysis based on Bray–Curtis dissimilarity clearly separated soda lake and rumen microbiomes along the first two principal coordinates axes (PERMANOVA *R*² = 0.338, *P* = 0.001; Fig. 1E).

### MAG recovery, novelty, and taxonomic composition

**Fig 2:**
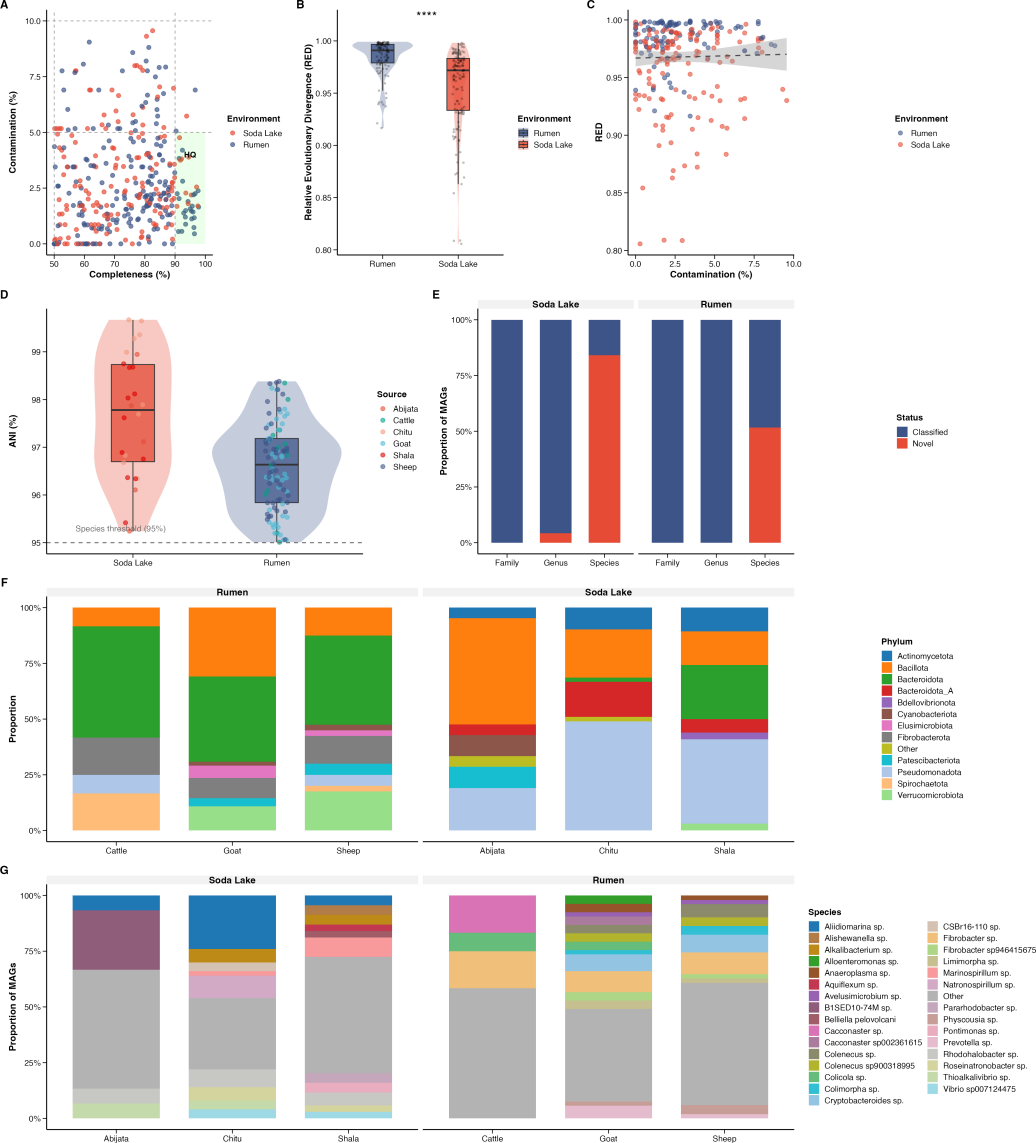
MAG quality, novelty, and taxonomic composition. (**A**) CheckM2 quality assessment (completeness vs. contamination). (**B**) RED distribution by environment. (**C**) RED vs. contamination. (**D**) ANI to the closest GTDB reference genome. (**E**) Taxonomic novelty by rank. (**F**) Phylum composition of MAGs by environment. (**G**) Top 20 most abundant species.

**Fig 3:**
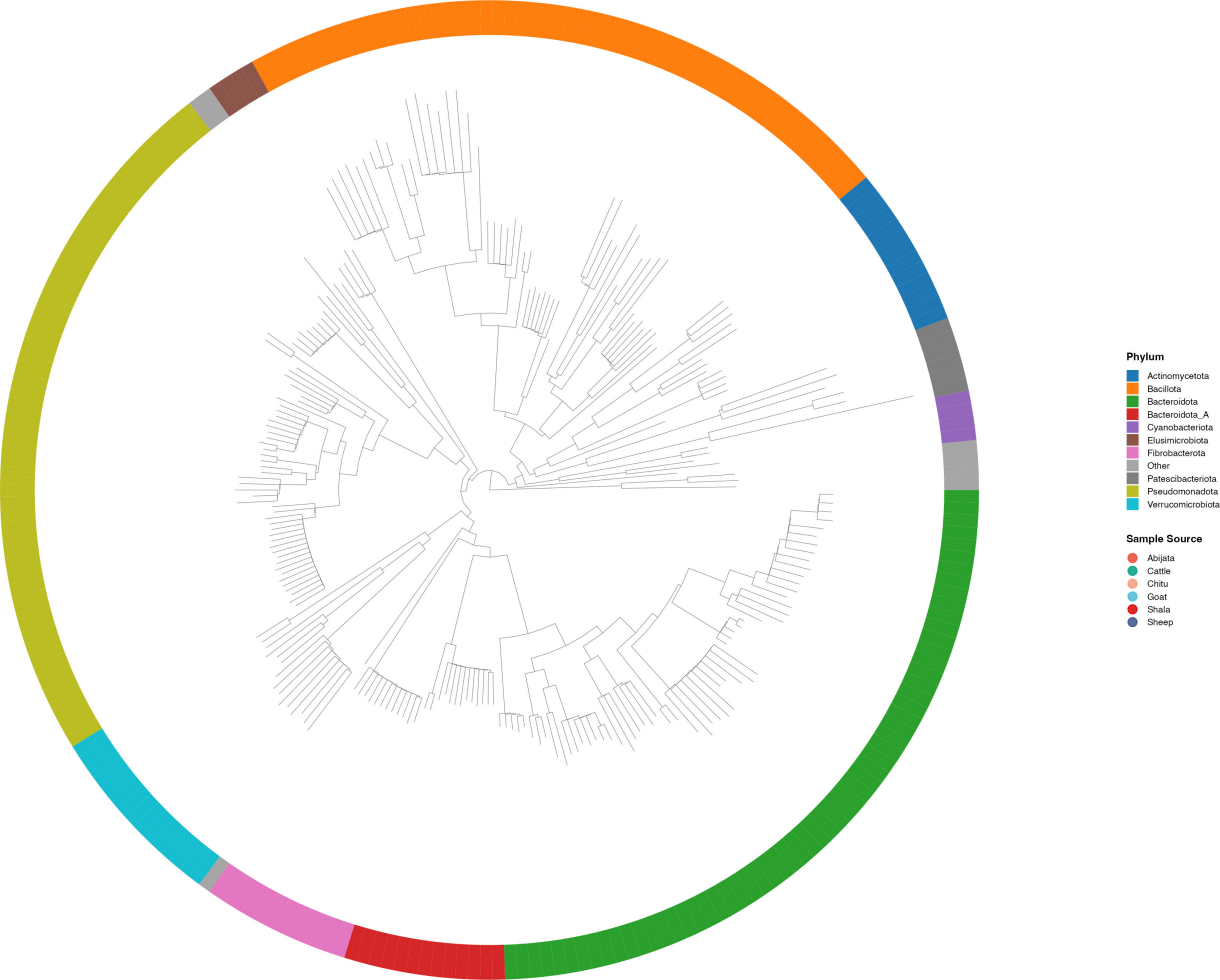
Circular phylogenomic tree of 245 dereplicated MAGs. Tips are colored by sample source (soda lakes: Abijata, Chitu, and Shala; rumen: cattle, goat, and sheep). The outer ring indicates phylum assignment from GTDB-Tk classification. Shape denotes environment (circle = soda lake, triangle = rumen).

A total of 371 quality-filtered medium-to-high-quality MAGs (≥50% completeness, ≤10 contamination) were retained after quality filtering with CheckM, comprising 164 from soda lakes and 207 from rumen samples (Fig. 2A). Of these, 34 MAGs (9.2%) met the stringent high-quality criteria (≥90% completeness, ≤5% contamination), including 10 from soda lakes (6.1%) and 24 from rumen (11.6%). Soda lake MAGs exhibited a significantly lower median relative evolutionary divergence (RED) value (Fig. 2B). Average nucleotide identity (ANI) to the closest GTDB reference genome was comparable between environments (soda lake median: 97.8%; rumen median: 96.6%; Fig. 2D). Taxonomic classification of MAGs using GTDB-Tk revealed striking differences in the proportion of novel lineages between the two ecosystems (Fig. 2E). Among soda lake MAGs, 84.1% (138 of 164) represented novel species lacking classified representatives in GTDB Release 220, compared with 51.7% (107 of 207) for rumen MAGs. At the genus level, 4.3% of soda lake MAGs were novel compared with 0% in the rumen. The phylogenomic tree constructed from dereplicated MAGs (Fig. 3) revealed distinct taxonomic profiles for each ecosystem. Rumen MAGs were dominated by *Bacteroidota*, *Bacillota*, *Verrucomicrobiota*, and *Fibrobacterota*. Soda lake MAGs were enriched in *Pseudomonadota*, *Bacillota*, *Bacteroidota*, and *Actinomycetota* (Fig. 2F). In rumen sources, the most frequently recovered classified species included *Fibrobacter* sp. and *Cryptobacteroides* sp. In soda lake sources, the most frequently recovered species included *Aliidiomarina* sp., *Natronospirillum* sp., and *Rhodohalobacter* sp. (Fig. 2G).

### Functional potential of the metagenome**-**assembled genomes (MAGs)

**Fig 4:**
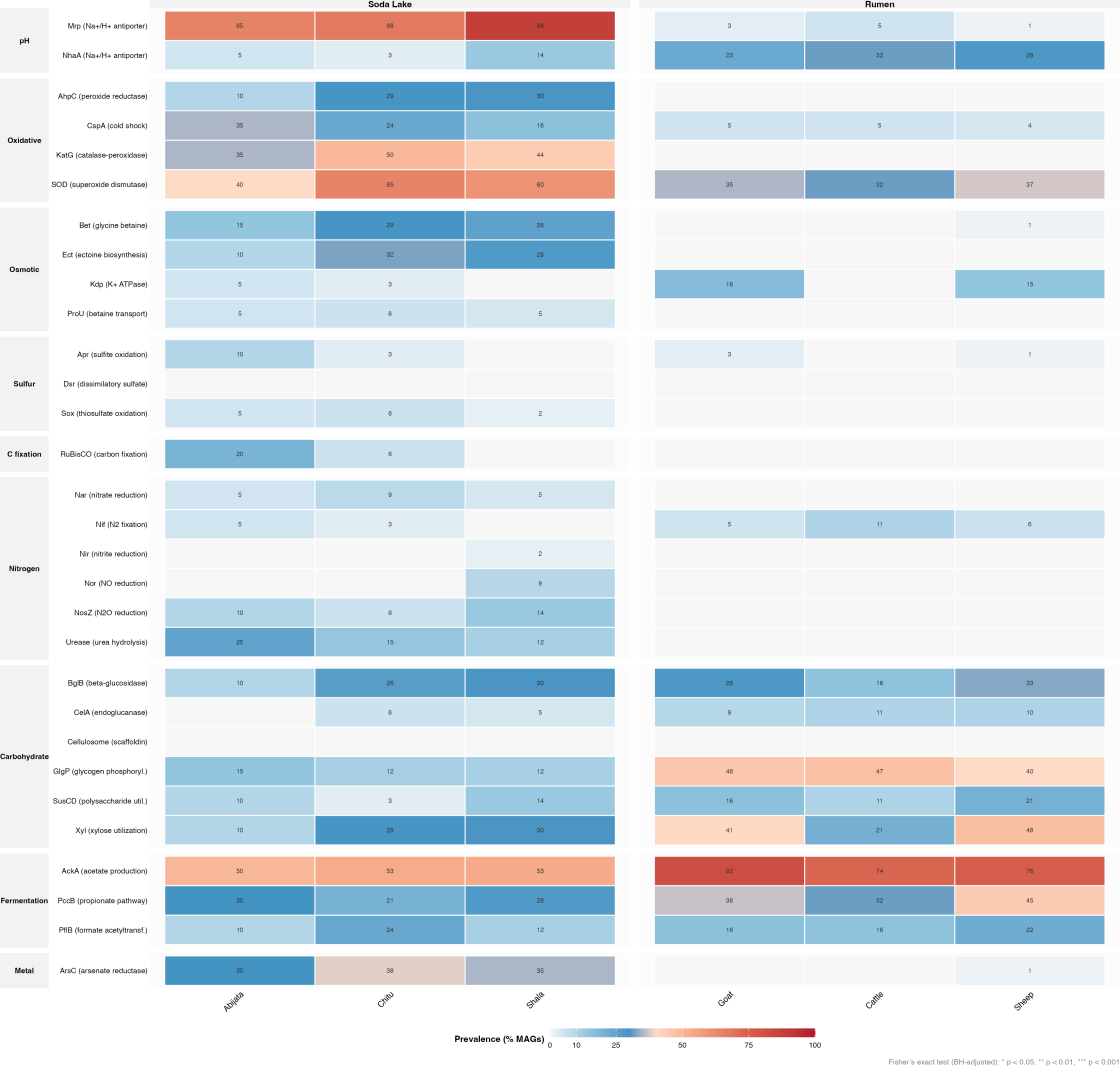
Functional pathway prevalence heatmap. Prevalence (percentage of MAGs carrying each pathway gene) displayed per sample source for soda lake and rumen Prokka-annotated MAGs across 29 metabolic pathways grouped into nine categories: pH homeostasis, oxidative stress, osmotic stress, sulfur cycling, carbon fixation, nitrogen cycling, carbohydrate catabolism, fermentation, and metal resistance. Enrichment assessed by Fisher’s exact test with BH correction.

Prokka-based gene annotation of MAGs and Fisher’s exact test with Benjamini–Hochberg (BH) adjustment across 29 manually curated pathways spanning nine functional groups revealed clear niche-specific functional signatures (Fig. 4). Soda lake MAGs were significantly enriched in pH homeostasis genes (Mrp Na^+^/H^+^ antiporter), oxidative stress defenses (KatG catalase-peroxidase, SOD superoxide dismutase), osmotic stress response (ectoine biosynthesis, glycine betaine synthesis, Kdp K^+^ ATPase), sulfur cycling (Sox thiosulfate oxidation, Apr sulfite oxidation), carbon fixation (RuBisCO), and nitrogen cycling (NosZ, urease). Rumen MAGs, in contrast, showed significantly higher prevalence of carbohydrate catabolism pathways (SusCD polysaccharide utilization, BglB β-glucosidase, xylose utilization, and glycogen phosphorylase), and fermentation genes (AckA acetate production and PccB propionate pathway), consistent with their specialized role in plant fiber degradation (Fig. 4).

### Carbohydrate-active enzyme diversity and sequence novelty

**Fig 5:**
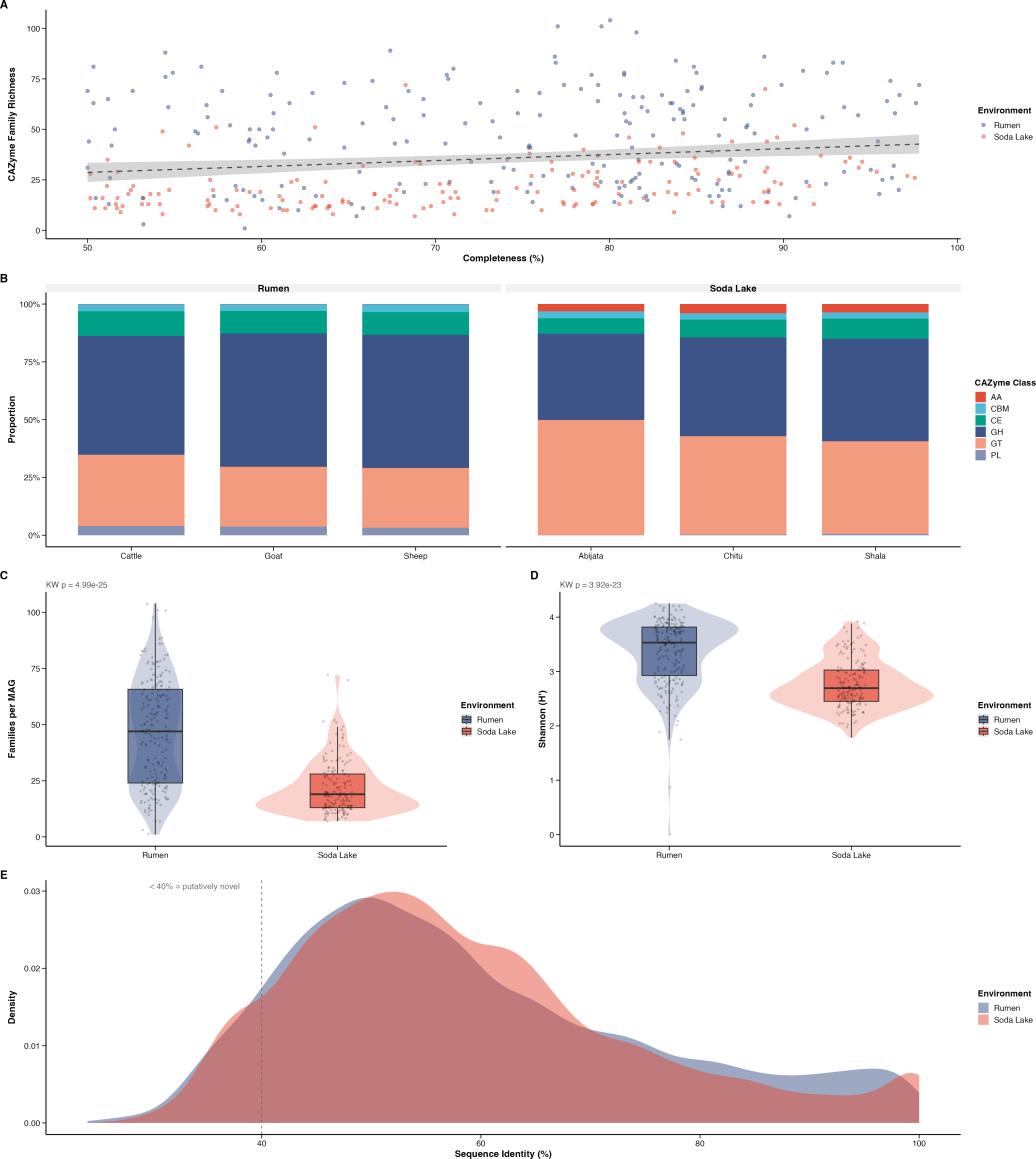
Carbohydrate-active enzyme diversity and novelty. (**A**) Correlation between MAG completeness and per-MAG CAZyme richness. (**B**) CAZyme class distribution across samples, showing relative contributions of GH, GT, PL, CE, AA, and CBM. (**C**) Per-MAG CAZyme richness, comparing soda lake and rumen environments. (**D**) Shannon diversity of CAZyme class composition per MAG. (**E**) DIAMOND identity density distribution showing sequence divergence of CAZymes from known references.

Carbohydrate-active enzyme (CAZyme) annotation yielded 26,541 predictions that were classified into functional classes: glycoside hydrolases (GH), glycosyltransferases (GT), polysaccharide lyases (PL), carbohydrate esterases (CE), auxiliary activities (AA), and carbohydrate-binding modules (CBM: Fig. 5). CAZyme richness positively correlated with MAG completeness (Fig. 5A; Spearman ρ = 0.225, *P* = 1.29 × 10⁻⁵). Among the classes, glycoside hydrolases (GH) and glycosyltransferases (GT) showed higher abundances in both soda lake and rumen ecosystems. CAZyme richness (Fig. 5C and D) was significantly higher in rumen samples (median 47) than in soda lakes. Sequence identity analysis via DIAMOND search against the CAZy database (Fig. 5E) showed that soda lake CAZymes had a broader distribution shifted toward lower sequence identity compared to rumen CAZymes.

### Glycoside hydrolase family repertoire and taxonomic contributors

**Fig 6:**
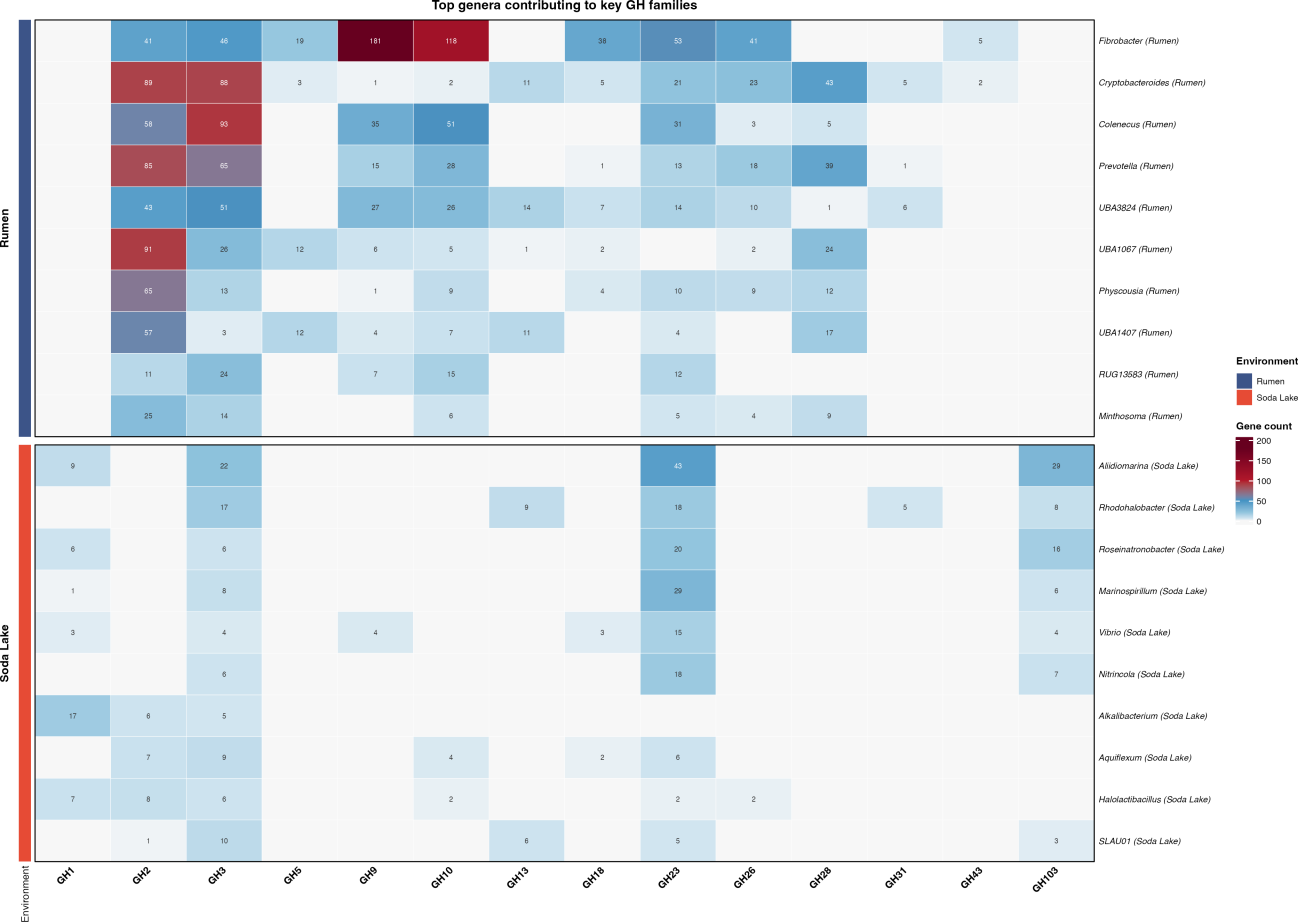
Glycoside hydrolase family repertoire across top genera and environments. Heatmap showing gene counts for 15 GH families (GH1, GH2, GH3, GH5, GH9, GH10, GH13, GH18, GH23, GH26, GH28, GH31, GH43, and GH103) across the top genera, split by environment (soda lakes vs. rumen). Tile size and color intensity reflect gene abundance.

Analysis of 14 key glycoside hydrolase (GH) families (GH1, GH2, GH3, GH5, GH9, GH10, GH13, GH18, GH23, GH26, GH28, GH31, GH43, and GH103) revealed distinct taxonomic and environmental patterns (Fig. 6). GH families showed separation between rumen and soda lake contributors. Rumen-associated genera, including *Fibrobacter*, *Prevotella*, and *Ruminococcus,* contributed the highest numbers of GH genes, particularly from families GH5, GH9, and GH10. Within rumen MAGs, *Fibrobacter* sp. accounted for the majority of GH5, GH9, and GH10 genes. Across environments, certain families (GH1, GH2, and GH3) were distributed among diverse taxa, while others (GH9 and GH10) were concentrated in fewer genera. In soda lakes, genera such as *Aliidiomarina*, *Rhodohalobacter*, *Marinospirillum*, and *Alkalibacterium* were the primary contributors, with GH23, GH103, GH13, and GH1 being the most abundant families (Fig. 6).

### Structural prediction and fold conservation

**Fig 7:**
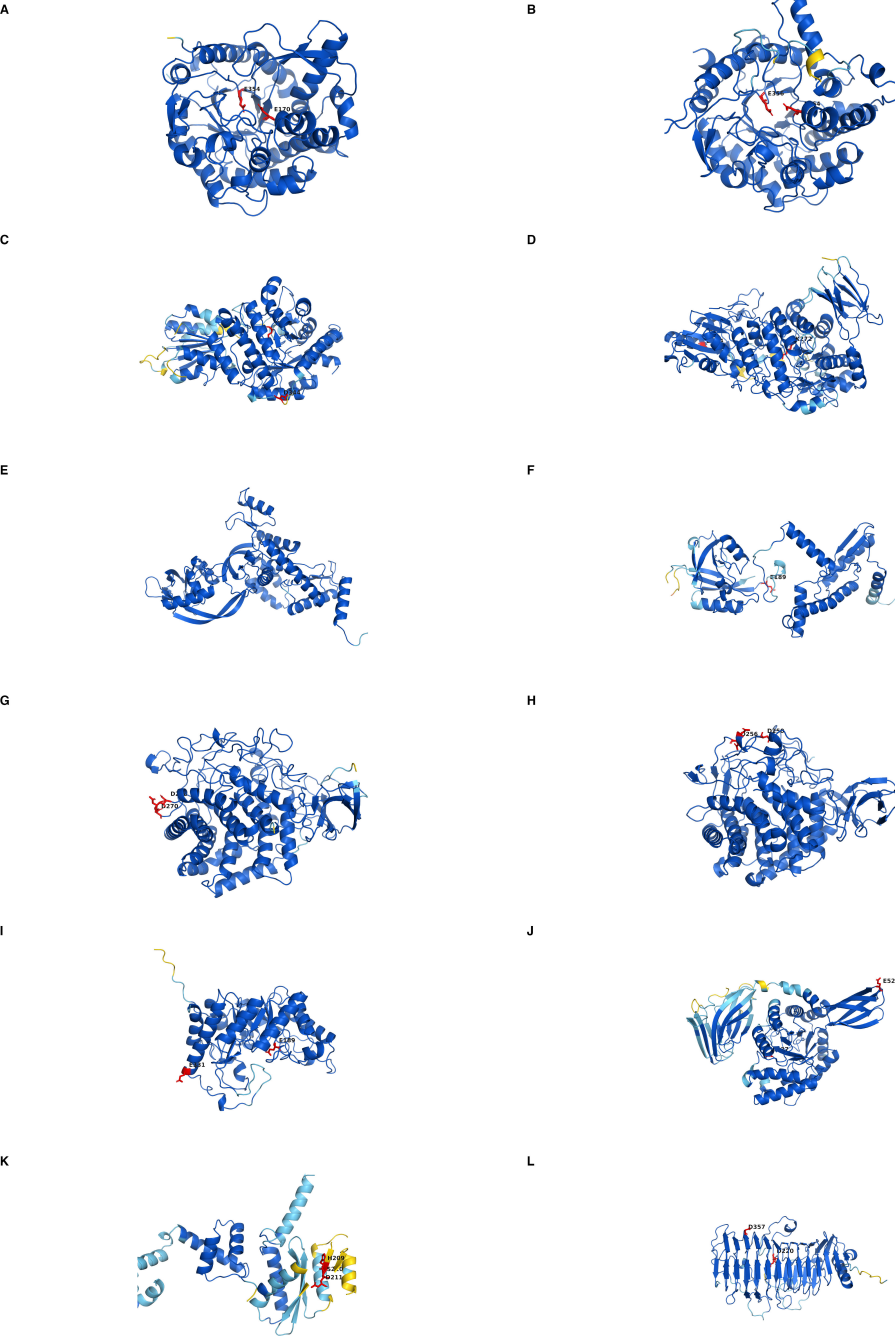
AlphaFold3-predicted structures of twelve CAZyme candidates. Models are colored by per-residue pLDDT confidence (blue >90, cyan 70–90, yellow 50–70, orange <50); catalytic residues are shown as red sticks and labeled. Columns correspond to the environment (left: Soda lake; right: Rumen) and rows to the glycoside hydrolase family. (**A**) GH1, soda lake; (**B**) GH1, rumen; (**C**) GH3, soda lake; (**D**) GH3, rumen; (**E**) GH5₁₁, soda lake; (**F**) GH5₁₁, rumen; (**G**) GH9, soda lake; (**H**) GH9, rumen; (**I**) GH10, soda lake; (**J**) GH10, rumen; (**K**) GH28, soda lake; and (**L**) GH28, rumen. All predictions used AlphaFold3 with default parameters; per-model metrics (pTM, mean pLDDT, fraction disordered) are reported in Table 1.

To investigate structural conservation of sequence-divergent CAZymes, we selected 12 representative proteins from six GH families (GH1, GH3, GH5_11, GH9, GH10, and GH28), one from each environment per family, and predicted their three-dimensional structures using AlphaFold3 (Table 1, Fig. 7). These candidates were drawn from taxonomically distinct MAGs with no phylum-level overlap between environments. Soda lake proteins originated from Pseudomonadota (*Roseinatronobacter* sp., *Pararhodobacter* sp.), Cyanobacteriota (*Limnospira fusiformis*), and Bacteroidota (CSBr16-58, *Aquiflexum* sp.), while rumen homologs were encoded by Bacillota (*Scatonaster* sp.), Fibrobacterota (*Fibrobacter* spp.), Verrucomicrobiota (UBA1067 sp.), and Bacteroidota (*Prevotella* sp.). These candidates spanned a wide range of sequence identities to characterize CAZy references (DIAMOND percent identity: 50.9%–100.0%). All 12 structures were predicted with high confidence (Table 1). Foldseek structural similarity searches against PDB100 revealed the extent of structural novelty in these enzymes. For 9 of 12 candidates, the closest whole-structure match in PDB100 was a genuine CAZyme from the same or a related GH family, confirming correct fold assignment. Active site analysis confirmed that catalytic residues were identified and conserved in 11 of 12 candidates. GH1 enzymes retained the Glu-Glu pair; GH3 the Lys/Asp catalytic dyad, and GH9 enzymes maintained the Asp-Asp-Asp catalytic triad (Table 1).

**TABLE 1:** AlphaFold3 structural predictions, dbCAN/DIAMOND CAZyme annotations, active-site conservation, and Foldseek PDB100 structural similarity for 12 representative CAZymes from soda lake and rumen metagenomes^*a*^

GH family	Env.	Source organism (phylum)	Diamond %ID	pTM	Mean pLDDT	Catalytic residues	Active site pLDDT	Top foldseek PDB hit	Lddt
GH1	Soda lake	Roseinatronobacter (Pseudomonadota)	97.5	0.97	98.5	Glu170, Glu354	99.0, 99.0	2J75 GH1 β-glucosidase (*T. maritima*)	0.86
GH1	Rumen	Scatonaster (Bacillota)	84.3	0.96	96.6	Glu164, Glu356	98.1, 98.7	4BCE GH1 β-glycosidase (*P. polymyxa*)	0.79
GH3	Soda lake	Limnospira (Cyanobacteriota)	100.0	0.94	92.6	Lys183, Asp344	97.6, 65.0	3SQL GH31 glycosidase (*Synechococcus* sp.)	0.84
GH3	Rumen	Fibrobacter (Fibrobacterota)	98.2	0.93	92.5	Lys272, Asp584	98.8, 97.3	5XXN GH3 β-glucosidase (*Fibrobacter* sp.)	0.69
GH5_11	Soda lake	CSBr16-58 (Bacteroidota)	50.9	0.90	96.8	Variant	N/A	1WKA Val-tRNA synthetase (*T. thermophilus*)*	0.84
GH5_11	Rumen	UBA1067 (Verrucomicrobiota)	52.9	0.75	93.5	Glu189	90.4	1GAX Val-tRNA synthetase (*T. thermophilus*)*	0.84
GH9	Soda lake	Limnospira (Cyanobacteriota)	100.0	0.93	94.3	Asp268, Asp269, Asp270	98.8, 98.5, 98.6	3X17 GH9 endoglucanase (metagenome)	0.66
GH9	Rumen	Fibrobacter (Fibrobacterota)	99.5	0.88	92.6	Asp250, Asp255, Asp256	97.3, 97.9, 97.4	6DHT GH9 endoglucanase (*Ruminiclostridium*)	0.78
GH10	Soda lake	Aquiflexum (Bacteroidota)	71.2	0.86	89.1	Glu189, Glu331	98.7, 98.0	4K68 GH10 xylanase (metagenome)	0.85
GH10	Rumen	Fibrobacter (Fibrobacterota)	99.1	0.84	90.0	Glu337, Glu522	97.4, 97.0	7D88 GH10 xylanase (*Bacillus* sp. KW1)	0.73
GH28	Soda lake	Pararhodobacter (Pseudomonadota)	67.2	0.76	75.3	His209, Ser210, Asp211	58.0, 53.4, 47.8	5EJK RSV integrase (*H. sapiens*)*	0.59
GH28	Rumen	Prevotella (Bacteroidota)	94.7	0.95	95.6	Asp220, Asp221, Asp357	96.1, 98.0, 98.0	3JUR GH28 exoPGase (*Bacteroides* sp.)	0.67

^*a*^ The Foldseek column shows the closest whole-structure match in PDB100; 9 of 12 proteins matched genuine CAZymes; the three non-CAZyme matches (GH5_11, soda GH28, marked with *) share ancestral fold scaffolds. Source organisms of PDB hits are shown in parentheses.

## DISCUSSION

Here, we compared the carbohydrate-degradation potential of two contrasting ecosystems, the Ethiopian soda lakes (alkaline, saline) and ruminant guts (anaerobic), using shotgun metagenomics, MAG recovery, CAZyme annotation, and AlphaFold3 structure prediction for 12 representative GH enzymes from six families. The goal was to assess whether structural and functional conservation of CAZymes persists despite extreme taxonomic and sequence divergence between these environments. Furthermore, the rumen microbiome represents the largest characterized metagenomic source of carbohydrate-active enzymes, and its unparalleled efficiency in degrading complex plant polymers positions it as a natural benchmark for biomass conversion. Soda lakes, despite their extreme alkalinity and salinity, sustain remarkably productive microbial communities with largely untapped enzymatic repertoires. Comparing the CAZyme profiles and functional potential of these two contrasting yet highly active ecosystems thus offers a unique opportunity to identify novel enzymes suited for biotechnological applications under industrially relevant conditions.

The microbial communities in the rumen and soda lakes exhibited distinct taxonomic profiles and diversity patterns that are likely shaped by their contrasting ecological roles and environmental conditions. Rumen samples displayed significantly higher microbial diversity compared to soda lake samples, consistent with the rumen’s function as a complex, cooperative digestive system where microbial consortia work synergistically to degrade plant material (44, 45). Beta-diversity analysis using principal coordinates analysis (PCoA) based on Bray-Curtis dissimilarity revealed a strong separation between rumen and soda lake microbiomes (PERMANOVA *P* = 0.001), confirming that microbial composition is primarily driven by environmental selection (46, 47).

One notable finding, consistent with our previous studies of Ethiopian soda lakes, was the striking discordance between read-level taxonomy and genome recovery for *Halomonas*. Despite its overwhelming dominance in MetaPhlAn4 profiles, *Halomonas* was not proportionally represented among the recovered MAGs. This likely reflects strain-level microdiversity causing assembly fragmentation, a well-recognized challenge in metagenomics whereby highly abundant organisms harboring numerous closely related strains produce chimeric contigs that resist binning into discrete genomic entities (48). This result underscores the importance of integrating read-based taxonomic profiling with genome-resolved metagenomics when studying under-characterized environments, as each approach captures complementary dimensions of community structure and function that the other misses.

Previous studies on the microbial composition and enzymatic potential of Ethiopia’s soda lakes have primarily relied on culture-dependent approaches, which capture only a limited subset of microbial diversity and functional capacity. Culture-independent surveys subsequently broadened this picture (9, 10), and our own recent amplicon-based studies showed that Ethiopian soda lakes harbor some of the highest prokaryotic and eukaryotic diversity recorded among extreme ecosystems. In line with this, comprehensive reviews of soda lake microbiology across the globe have established these ecosystems as globally significant biodiversity hotspots (6, 14, 49). A recent biogeographic analysis of 51 soda lake metagenomes across four continents identified 1,217 region-specific taxa (50), positioning Ethiopian lakes as a particularly under-explored fraction of this global diversity. Moreover, although several studies have characterized polysaccharide-degrading enzymes from global soda lakes, including cellulases, hemicellulases, and chitinases (51–53), genome-resolved, structurally validated CAZyme catalogs from Ethiopian soda lakes have been lacking. The present study begins to close that gap by coupling MAG recovery with AlphaFold3-based structural prediction of candidate enzymes.

The substantial proportion of unclassified microbial reads and the broader RED distribution observed in soda lake MAGs underscore the presence of novel, evolutionarily distinct microbial lineages in these environments. This finding aligns with studies indicating that soda lakes harbor diverse microbial communities with a significant fraction of unclassified taxa (54, 55). In contrast, the rumen microbiome exhibits a more constrained RED range, reflecting a higher degree of relatedness to known microbial taxa, consistent with its well-characterized nature and functional specialization in plant polysaccharide degradation (13, 56). This pattern suggests that soda lakes serve as evolutionary hotspots for microbial novelty, while the rumen microbiome maintains functional stability through conserved microbial lineages. Complementary enrichment-based approaches could further enhance the recovery of rare but functionally important taxa from these environments; our previous amplicon-based study of Ethiopian soda lakes (18) demonstrated the potential of selective enrichment cultures for capturing cultivable diversity not detected by direct sequencing alone. Recent studies combining enrichment with environmental analysis of airborne microbial communities (57) have strengthened the value of integrating culture-dependent and culture-independent strategies, although with the caveat that enrichment inherently biases recovery toward cultivable lineages.

The rumen microbiome was dominated by *Bacteroidota*, *Fibrobacterota*, and *Bacillota* (*Firmicutes*) at the phylum level (Fig. 1A), and *Fibrobacter*, *Cryptobacteroides*, and *Prevotella* at the genus level (Fig. 2G). Among the key glycoside hydrolase families, *Fibrobacter* and *Prevotella* were the principal contributors to GH5, GH9, and GH10 repertoires, accounting for the majority of cellulase and xylanase genes in the rumen (Fig. 5). These taxa are known for their ability to break down complex carbohydrates derived from plant material, contributing to host nutrition and energy production through fermentation processes (58–61). *Fibrobacter,* being a primary cellulose degrader, and *Prevotella* contribute significantly to hemicellulose breakdown and overall carbohydrate metabolism (62–65). In contrast, soda lake communities were dominated by *Pseudomonadota*, with lesser contributions from *Bacillota* (*Firmicutes*) and *Cyanobacteriota* (Fig. 1A), a taxonomic architecture consistent with previous characterizations of East African soda lake microbiomes (7, 49, 66). The most frequently recovered soda lake genera, *Aliidiomarina*, *Rhodohalobacter*, *Marinospirillum*, and *Alkalibacterium* (Fig. 2G), are characterized by their adaptations to hypersaline and alkaline conditions (67–70). These taxa were also responsible for a functionally distinct set of GH families, including GH23 (lysozyme), GH103 (lytic transglycosylase), GH13 (α-amylase), and GH1 (β-glucosidase) (Fig. 6). This distributional pattern underscores how different ecological niches select for different carbohydrate-processing strategies and taxonomic distributions.

Another striking result we found was that soda lake MAGs were notably enriched in genes encoding proteins for metabolic processes required for survival in extreme conditions (71–74). Our expanded pathway screening across 29 gene sets and 9 functional groups (Fig. 4) confirmed and extended these findings. Soda lake MAGs were significantly enriched in pH homeostasis machinery (Mrp Na^+^/H^+^ antiporter, present in 65%–88% of soda lake MAGs versus ≤ 5% in rumen), oxidative stress defenses (KatG catalase-peroxidase, SOD superoxide dismutase), osmolyte biosynthesis (ectoine, glycine betaine), sulfur cycling (Sox thiosulfate oxidation, Apr sulfite oxidation), carbon fixation (RuBisCO), nitrogen cycling (NosZ, urease), and arsenate resistance (ArsC). In contrast, rumen MAGs showed significantly higher prevalence of carbohydrate catabolism genes (SusCD polysaccharide utilization, GlgP glycogen phosphorylase, xylose utilization) and short-chain fatty acid fermentation pathways (AckA acetate production, PccB propionate pathway), reflecting the rumen’s specialized role as a plant-fiber fermentation chamber (75).

The structural analysis showed the conservation of protein fold architecture across taxonomically unrelated MAGs from soda lakes and rumen. All 12 AlphaFold3-predicted structures adopted canonical GH family folds with high confidence (pTM0.75–0.97) (76), and Foldseek structural comparison confirmed that 9 of 12 predictions matched genuine CAZymes of the same or closely related GH family in the PDB100 database (Table 1; Fig. 7). In addition, despite the differences in the environments, the predicted structures were consistent with the principle that TIM-barrel (GH1, GH5, and GH10) and parallel β-helix (GH28) folding (77–79). Active-site residues were conserved in 23 of 26 annotated positions, with per-residue pLDDT scores of 65–99 at catalytic sites across eleven structures. GH1 enzymes retained the expected Glu acid/base and Glu nucleophile pair required for retaining mechanism catalysis (79); GH3 enzymes conserved the Lys/Asp catalytic dyad (80); GH9 enzymes maintained the Asp-Asp-Asp catalytic triad (81); GH10 enzymes retained the Glu acid/base catalyst and Glu nucleophile (82); and the rumen GH28 candidate conserved the three-aspartate catalytic triad (83) (Asp220, Asp221, and Asp357) characteristic of the inverting mechanism. However, in our active site annotation, the soda lake GH28 pectinase exhibited among the lowest confidence scores (pTM 0.76, Foldseek LDDT 0.595, active-site pLDDT 47.8–58.0) and substitutions at two of three canonical catalytic positions (His209 and Ser210 replacing the canonical Asp–Asp motif), suggesting that this might be a novel catalytic mechanism and the soda lake GH5_11 endoglucanase lacked the canonical catalytic glutamate retained by its rumen homolog (Glu189).

We picked the six GH families, GH1, GH3, GH5, GH9, GH10, and GH28, for structural prediction because of their established roles in lignocellulosic biomass degradation, collectively covering the hydrolysis of cellulose, hemicellulose, and pectin. The recovery of structurally conserved, catalytically intact representatives from both haloalkaliphilic soda lake and rumen metagenomes highlights the potential of extremophile-derived CAZymes as industrial biocatalysts capable of operating under elevated temperature, non-neutral pH, high ionic strength, and product inhibition (84, 85). Our recent characterization of the soda lake GH3 β-glucosidase CelGH3_f17, which exhibits optimal activity at pH 8.5, retains activity at 4 M NaCl, and is glucose-stimulated up to 300 mM, exemplifies how such extremophilic enzymes can address key bottlenecks in biomass saccharification (17). The structural predictions reported here demonstrate that the catalytic architecture underlying this functionality is preserved at the three-dimensional level even across taxonomically unrelated donors. Heterologous expression, biochemical characterization, and directed evolution of the most promising candidates will be essential to translate these predictions into verified biotechnological tools, positioning soda lake and rumen metagenomes as complementary and largely untapped reservoirs of industrially relevant CAZyme diversity.

### Conclusion

This study provides the first integrated comparison of carbohydrate-degradation potential between Ethiopian soda lake and ruminant gut metagenomes, combining genome-resolved metagenomics with AlphaFold3 structural prediction. From 371 quality-filtered MAGs, we show that the rumen microbiome functions as a specialized, high-diversity fiber-degradation system dominated by Bacteroidota and Fibrobacterota, whereas soda lake communities harbor greater evolutionary novelty and are enriched in extremophile-specific stress response and biogeochemical cycling pathways. All predicted structures adopted canonical folds with high confidence (pTM 0.75–0.97) and conserved catalytic residues in 23 of 26 active-site positions, demonstrating that three-dimensional enzyme architecture is maintained across radically different environments and taxonomic lineages. These findings, together with our recent characterization of the alkaline-, salt-, and glucose-tolerant soda lake GH3 β-glucosidase CelGH3_f17, establish soda lake and rumen metagenomes as complementary reservoirs of industrially relevant CAZyme diversity. Heterologous expression, biochemical characterization, and directed evolution of the most promising candidates identified here will be the next essential steps toward translating these structural predictions into verified biotechnological tools for biomass conversion under extreme industrial conditions.

## Data Availability

All raw sequencing data have been deposited in the NCBI Sequence Read Archive (SRA) under BioProject accession number PRJNA1273195. Additional data supporting the findings of this study are available from the corresponding author upon reasonable request. Custom scripts used for data preprocessing, taxonomy and functional annotation, statistical analysis, and visualization are available at https://github.com/OliyadJe/Soda-Lakes-and-Rumen-Metagenomics.

## SUPPLEMENTAL MATERIAL

Figure S1 msystems.00485-26-s0001.png Assessment of metagenomic sequencing coverage using Nonpareil curves.

Legend msystems.00485-26-s0002.docx Supplemental figure legend.

Table S1 msystems.00485-26-s0003.xlsx Metadata for soda lake and rumen microbiome samples.

ASM does not own the copyrights to Supplemental Material that may be linked to, or accessed through, an article. The authors have granted ASM a non-exclusive, world-wide license to publish the Supplemental Material files. Please contact the corresponding author directly for reuse.

## References

[1] Barrett K, Lange L, Børsting CF, Olijhoek DW, Lund P, Meyer AS. 2022. Changes in the metagenome-encoded CAZymes of the rumen microbiome are linked to feed-induced reductions in methane emission from holstein cows. Front Microbiol 13:855590. doi:10.3389/fmicb.2022.855590 35668758 PMC9163818

[2] Hinsu AT, Tulsani NJ, Panchal KJ, Pandit RJ, Jyotsana B, Dafale NA, Patil NV, Purohit HJ, Joshi CG, Jakhesara SJ. 2021. Characterizing rumen microbiota and CAZyme profile of Indian dromedary camel (Camelus dromedarius) in response to different roughages. Sci Rep 11:9400. doi:10.1038/s41598-021-88943-9 33931716 PMC8087840

[3] Auer E, Lazuka A, Huguenin-Bizot B, Jehmlich N, Déjean S, Lombard V, Henrissat B, O’Donohue M, Hernandez-Raquet G. 2023. Horizontal metaproteomics and CAZymes analysis of lignocellulolytic microbial consortia selectively enriched from cow rumen and termite gut. ISME Communications 3:1–12. doi:10.1038/s43705-023-00339-037081121

[4] Jeilu O, Simachew A, Alexandersson E, Johansson E, Gessesse A. 2022. Discovery of novel carbohydrate degrading enzymes from soda lakes through functional metagenomics. Front Microbiol 13:1059061. doi:10.3389/fmicb.2022.1059061 36569080 PMC9768486

[5] Liang J, Zhang R, Chang J, Chen L, Nabi M, Zhang H, Zhang G, Zhang P. 2024. Rumen microbes, enzymes, metabolisms, and application in lignocellulosic waste conversion - a comprehensive review. Biotechnol Adv 71:108308. doi:10.1016/j.biotechadv.2024.108308 38211664

[6] Sorokin D. Y., Banciu HL, Muyzer G. 2015. Functional microbiology of soda lakes. Curr Opin Microbiol 25:88–96. doi:10.1016/j.mib.2015.05.004 26025021

[7] Sorokin Dimitry Y, Berben T, Melton ED, Overmars L, Vavourakis CD, Muyzer G. 2014. Microbial diversity and biogeochemical cycling in soda lakes. Extremophiles 18:791–809. doi:10.1007/s00792-014-0670-9 25156418 PMC4158274

[8] Agembe S, Ojwang W, Olilo C, Omondi R, Ongore C. 2017. Soda lakes of the Rift Valley (Kenya). *In* Finlayson CM, Milton GR, Prentice RC, Davidson NC (ed), The wetland book: II: distribution, description and conservation. Springer Netherlands.

[9] Simachew A, Lanzén A, Gessesse A, Øvreås L. 2016. Prokaryotic community diversity along an increasing salt gradient in a soda ash concentration pond. Microb Ecol 71:326–338. doi:10.1007/s00248-015-0675-7 26408190

[10] Lanzén A, Simachew A, Gessesse A, Chmolowska D, Jonassen I, Øvreås L. 2013. Surprising prokaryotic and eukaryotic diversity, community structure and biogeography of Ethiopian soda lakes. PLoS One 8:e72577. doi:10.1371/journal.pone.0072577 24023625 PMC3758324

[11] Cammack KM, Austin KJ, Lamberson WR, Conant GC, Cunningham HC. 2018. Ruminant nutrition symposium: tiny but mighty: the role of the rumen microbes in livestock production. J Anim Sci 96:752–770. doi:10.1093/jas/skx053 29385535 PMC6140983

[12] Matthews C, Crispie F, Lewis E, Reid M, O’Toole PW, Cotter PD. 2019. The rumen microbiome: a crucial consideration when optimising milk and meat production and nitrogen utilisation efficiency. Gut Microbes 10:115–132. doi:10.1080/19490976.2018.1505176 30207838 PMC6546327

[13] Stewart RD, Auffret MD, Warr A, Walker AW, Roehe R, Watson M. 2019. Compendium of 4,941 rumen metagenome-assembled genomes for rumen microbiome biology and enzyme discovery. Nat Biotechnol 37:953–961. doi:10.1038/s41587-019-0202-3 31375809 PMC6785717

[14] Zorz JK, Sharp C, Kleiner M, Gordon PMK, Pon RT, Dong X, Strous M. 2019. A shared core microbiome in soda lakes separated by large distances. Nat Commun 10:4230. doi:10.1038/s41467-019-12195-5 31530813 PMC6748926

[15] Pellegrinetti TA, Cotta SR, Feitosa YB, Melo PLA, Bieluczyk W, Silva AMM, Mendes LW, Sarmento H, Camargo PB, Tsai SM, et al.. 2024. The role of microbial communities in biogeochemical cycles and greenhouse gas emissions within tropical soda lakes. Sci Total Environ 947:174646. doi:10.1016/j.scitotenv.2024.174646 38986696

[16] Gheibipour M, et al.. 2025. Bioengineering the rumen microbiota as an advanced biocatalyst for renewable fuels. *In* Dar MA, Zabed HM, Shahnawaz M (ed), Recent trends in lignocellulosic biofuels and bioenergy: advancements and sustainability assessment. Springer Nature Singapore.

[17] Jeilu O, Alexandersson E, Johansson E, Simachew A, Gessesse A. 2024. A novel GH3-β-glucosidase from soda lake metagenomic libraries with desirable properties for biomass degradation. Sci Rep 14:10012. doi:10.1038/s41598-024-60645-y 38693138 PMC11063200

[18] Jeilu O, Gessesse A, Simachew A, Johansson E, Alexandersson E. 2022. Prokaryotic and eukaryotic microbial diversity from three soda lakes in the East African Rift Valley determined by amplicon sequencing. Front Microbiol 13:999876. doi:10.3389/fmicb.2022.999876 36569062 PMC9772273

[19] Robinson SL, Piel J, Sunagawa S. 2021. A roadmap for metagenomic enzyme discovery. Nat Prod Rep 38:1994–2023. doi:10.1039/d1np00006c 34821235 PMC8597712

[20] Mathieu E, Léjard V, Ezzine C, Govindin P, Morat A, Giat M, Lapaque N, Doré J, Blottière HM. 2023. An insight into functional metagenomics: a high-throughput approach to decipher food-microbiota-host interactions in the human gut. Int J Mol Sci 24:17630. doi:10.3390/ijms242417630 38139456 PMC10744307

[21] Zuñiga C, Zaramela L, Zengler K. 2017. Elucidation of complexity and prediction of interactions in microbial communities. Microb Biotechnol 10:1500–1522. doi:10.1111/1751-7915.12855 28925555 PMC5658597

[22] Usyk M, Peters BA, Karthikeyan S, McDonald D, Sollecito CC, Vazquez-Baeza Y, Shaffer JP, Gellman MD, Talavera GA, Daviglus ML, et al.. 2023. Comprehensive evaluation of shotgun metagenomics, amplicon sequencing, and harmonization of these platforms for epidemiological studies. Cell Rep Methods 3:100391. doi:10.1016/j.crmeth.2022.100391 36814836 PMC9939430

[23] Andrews S. 2010. FastQC. Babraham Bioinformatics.

[24] Bolger AM, Lohse M, Usadel B. 2014. Trimmomatic: a flexible trimmer for Illumina sequence data. Bioinformatics 30:2114–2120. doi:10.1093/bioinformatics/btu170 24695404 PMC4103590

[25] Ewels P, Magnusson M, Lundin S, Käller M. 2016. MultiQC: summarize analysis results for multiple tools and samples in a single report. Bioinformatics 32:3047–3048. doi:10.1093/bioinformatics/btw354 27312411 PMC5039924

[26] Rodriguez-R LM, Konstantinidis KT. 2014. Nonpareil: a redundancy-based approach to assess the level of coverage in metagenomic datasets. Bioinformatics 30:629–635. doi:10.1093/bioinformatics/btt584 24123672

[27] Blanco-Míguez A, Beghini F, Cumbo F, McIver LJ, Thompson KN, Zolfo M, Manghi P, Dubois L, Huang KD, Thomas AM, et al.. 2023. Extending and improving metagenomic taxonomic profiling with uncharacterized species using MetaPhlAn 4. Nat Biotechnol 41:1633–1644. doi:10.1038/s41587-023-01688-w 36823356 PMC10635831

[28] Li D, Liu CM, Luo R, Sadakane K, Lam TW. 2015. MEGAHIT: an ultra-fast single-node solution for large and complex metagenomics assembly via succinct de Bruijn graph. Bioinformatics 31:1674–1676. doi:10.1093/bioinformatics/btv033 25609793

[29] Gurevich A, Saveliev V, Vyahhi N, Tesler GQ. 2013. QUAST: quality assessment tool for genome assemblies. Bioinformatics 29:1072–1075. doi:10.1093/bioinformatics/btt086 23422339 PMC3624806

[30] Kang DD, Li F, Kirton E, Thomas A, Egan R, An H, Wang Z. 2019. MetaBAT 2: an adaptive binning algorithm for robust and efficient genome reconstruction from metagenome assemblies. PeerJ 7:e7359. doi:10.7717/peerj.7359 31388474 PMC6662567

[31] Langmead B, Salzberg SL. 2012. Fast gapped-read alignment with Bowtie 2. Nat Methods 9:357–359. doi:10.1038/nmeth.1923 22388286 PMC3322381

[32] Danecek P, Bonfield JK, Liddle J, Marshall J, Ohan V, Pollard MO, Whitwham A, Keane T, McCarthy SA, Davies RM, et al.. 2021. Twelve years of SAMtools and BCFtools. Gigascience 10:giab008. doi:10.1093/gigascience/giab008 33590861 PMC7931819

[33] Parks DH, Imelfort M, Skennerton CT, Hugenholtz P, Tyson GW. 2015. CheckM: assessing the quality of microbial genomes recovered from isolates, single cells, and metagenomes. Genome Res 25:1043–1055. doi:10.1101/gr.186072.114 25977477 PMC4484387

[34] Olm MR, Brown CT, Brooks B, Banfield JF. 2017. dRep: a tool for fast and accurate genomic comparisons that enables improved genome recovery from metagenomes through de-replication. ISME J 11:2864–2868. doi:10.1038/ismej.2017.126 28742071 PMC5702732

[35] Chaumeil PA, Mussig AJ, Hugenholtz P, Parks DH. 2020. GTDB-Tk: a toolkit to classify genomes with the Genome Taxonomy Database. Bioinformatics 36:1925–1927. doi:10.1093/bioinformatics/btz848 PMC7703759 31730192

[36] Asnicar F, Thomas AM, Beghini F, Mengoni C, Manara S, Manghi P, Zhu Q, Bolzan M, Cumbo F, May U, et al.. 2020. Precise phylogenetic analysis of microbial isolates and genomes from metagenomes using PhyloPhlAn 3.0. Nat Commun 11:2500. doi:10.1038/s41467-020-16366-7 32427907 PMC7237447

[37] Seemann T. 2014. Prokka: rapid prokaryotic genome annotation. Bioinformatics 30:2068–2069. doi:10.1093/bioinformatics/btu153 24642063

[38] Lombard V, Golaconda Ramulu H, Drula E, Coutinho PM, Henrissat B. 2014. The carbohydrate-active enzymes database (CAZy) in 2013. Nucleic Acids Res 42:D490–5. doi:10.1093/nar/gkt1178 24270786 PMC3965031

[39] Abramson J, Adler J, Dunger J, Evans R, Green T, Pritzel A, Ronneberger O, Willmore L, Ballard AJ, Bambrick J, et al.. 2024. Accurate structure prediction of biomolecular interactions with AlphaFold 3. Nature 630:493–500. doi:10.1038/s41586-024-07487-w 38718835 PMC11168924

[40] Rosignoli S, Paiardini A. 2022. Boosting the full potential of PyMOL with structural biology plugins. Biomolecules 12:1764. doi:10.3390/biom12121764 36551192 PMC9775141

[41] van Kempen M, Kim SS, Tumescheit C, Mirdita M, Lee J, Gilchrist CLM, Söding J, Steinegger M. 2024. Fast and accurate protein structure search with Foldseek. Nat Biotechnol 42:243–246. doi:10.1038/s41587-023-01773-0 37156916 PMC10869269

[42] Chen Y, Zhang H, Wang W, Shen Y, Ping Z. 2024. Rapid generation of high-quality structure figures for publication with PyMOL-PUB. Bioinformatics 40:btae139. doi:10.1093/bioinformatics/btae139 38449297 PMC10950480

[43] R Core Team. 2023. R: A language and environment for statistical computing. R Foundation for Statistical Computing, Vienna, Austria.

[44] Wu Y, Jiao C, Diao Q, Tu Y. 2023. Effect of dietary and age changes on ruminal microbial diversity in holstein calves. Microorganisms 12:12. doi:10.3390/microorganisms12010012 38276181 PMC10818949

[45] Huws SA, Creevey CJ, Oyama LB, Mizrahi I, Denman SE, Popova M, Muñoz-Tamayo R, Forano E, Waters SM, Hess M, et al.. 2018. Addressing global ruminant agricultural challenges through understanding the rumen microbiome: past, present, and future. Front Microbiol 9:2161. doi:10.3389/fmicb.2018.02161 30319557 PMC6167468

[46] Yan L, Herrmann M, Kampe B, Lehmann R, Totsche KU, Küsel K. 2020. Environmental selection shapes the formation of near-surface groundwater microbiomes. Water Res 170:115341. doi:10.1016/j.watres.2019.115341 31790889

[47] Junkins EN, McWhirter JB, McCall L-I, Stevenson BS. 2022. Environmental structure impacts microbial composition and secondary metabolism. ISME Commun 2:15. doi:10.1038/s43705-022-00097-5 37938679 PMC9723690

[48] Lapidus AL, Korobeynikov AI. 2021. Metagenomic data assembly - the way of decoding unknown microorganisms. Front Microbiol 12:613791. doi:10.3389/fmicb.2021.613791 33833738 PMC8021871

[49] Antony CP, Kumaresan D, Hunger S, Drake HL, Murrell JC, Shouche YS. 2013. Microbiology of Lonar lake and other soda lakes. ISME J 7:468–476. doi:10.1038/ismej.2012.137 23178675 PMC3578565

[50] Ren M, Wang J. 2025. Biogeography of soda lake microbiome and uneven cross-continent transition rates. Front Microbiol 16:1614302. doi:10.3389/fmicb.2025.1614302 40778207 PMC12330390

[51] Sorokin DY, Tourova TP, Sukhacheva MV, Mardanov AV, Ravin NV. 2012. Bacterial chitin utilisation at extremely haloalkaline conditions. Extremophiles 16:883–894. doi:10.1007/s00792-012-0484-6 23007247

[52] Grum-Grzhimaylo AA, Falkoski DL, van den Heuvel J, Valero-Jiménez CA, Min B, Choi I-G, Lipzen A, Daum CG, Aanen DK, Tsang A, et al.. 2018. The obligate alkalophilic soda-lake fungus Sodiomyces alkalinus has shifted to a protein diet. Mol Ecol 27:4808–4819. doi:10.1111/mec.14912 30368956

[53] Sorokin DY, Merkel AY, Khizhniak TV. 2024. Isolation and characterization of cellulose-mineralizing haloalkaliphilic bacteria from Siberian soda lakes. Front Microbiol 15:1523074. doi:10.3389/fmicb.2024.1523074 39764452 PMC11700989

[54] Vavourakis CD, Ghai R, Rodriguez-Valera F, Sorokin DY, Tringe SG, Hugenholtz P, Muyzer G. 2016. Metagenomic insights into the uncultured diversity and physiology of microbes in four hypersaline soda lake brines. Front Microbiol 7:211. doi:10.3389/fmicb.2016.00211 26941731 PMC4766312

[55] Andreote APD, Dini-Andreote F, Rigonato J, Machineski GS, Souza BCE, Barbiero L, Rezende-Filho AT, Fiore MF. 2018. Contrasting the genetic patterns of microbial communities in soda lakes with and without cyanobacterial bloom. Front Microbiol 9:244. doi:10.3389/fmicb.2018.00244 29520256 PMC5827094

[56] Stergiadis S, Cabeza-Luna I, Mora-Ortiz M, Stewart RD, Dewhurst RJ, Humphries DJ, Watson M, Roehe R, Auffret MD. 2020. Unravelling the role of rumen microbial communities, genes, and activities on milk fatty acid profile using a combination of omics approaches. Front Microbiol 11:590441. doi:10.3389/fmicb.2020.590441 33552010 PMC7859430

[57] Jeilu O, Sumner JT, Moghadam AA, Thompson KN, Huttenhower C, Catlett C, Hartmann EM. 2025. Metagenomic profiling of airborne microbial communities from aircraft filters and face masks. Microbiome 13:249. doi:10.1186/s40168-025-02276-7 41340070 PMC12676802

[58] Pereira AM, de Lurdes Nunes Enes Dapkevicius M, Borba AES. 2022. Alternative pathways for hydrogen sink originated from the ruminal fermentation of carbohydrates: which microorganisms are involved in lowering methane emission? Anim Microbiome 4:5. doi:10.1186/s42523-021-00153-w 34991722 PMC8734291

[59] Comtet-Marre S, Parisot N, Lepercq P, Chaucheyras-Durand F, Mosoni P, Peyretaillade E, Bayat AR, Shingfield KJ, Peyret P, Forano E. 2017. Metatranscriptomics reveals the active bacterial and eukaryotic fibrolytic communities in the rumen of dairy cow fed a mixed diet. Front Microbiol 8:67. doi:10.3389/fmicb.2017.00067 28197133 PMC5281551

[60] Gharechahi J, Sarikhan S, Han J-L, Ding X-Z, Salekdeh GH. 2022. Functional and phylogenetic analyses of camel rumen microbiota associated with different lignocellulosic substrates. NPJ Biofilms Microbiomes 8:46. doi:10.1038/s41522-022-00309-9 35676509 PMC9177762

[61] Chai J, Zhuang Y, Cui K, Bi Y, Zhang N. 2024. Metagenomics reveals the temporal dynamics of the rumen resistome and microbiome in goat kids. Microbiome 12:14. doi:10.1186/s40168-023-01733-5 38254181 PMC10801991

[62] Takizawa S, Asano R, Abe K, Fukuda Y, Baba Y, Sakurai R, Tada C, Nakai Y. 2023. Relationship between rumen microbial composition and fibrolytic isozyme activity during the biodegradation of rice straw powder using rumen fluid. Microbes Environ 38:ME23041. doi:10.1264/jsme2.ME23041 37766554 PMC10522846

[63] Dao T-K, Do T-H, Le N-G, Nguyen H-D, Nguyen T-Q, Le T-T-H, Truong N-H. 2021. Understanding the role of Prevotella genus in the digestion of lignocellulose and other substrates in vietnamese native goats’ rumen by metagenomic deep sequencing. Animals (Basel) 11:3257. doi:10.3390/ani11113257 34827987 PMC8614338

[64] Betancur-Murillo CL, Aguilar-Marín SB, Jovel J. 2022. Prevotella: a key player in ruminal metabolism. Microorganisms 11:1. doi:10.3390/microorganisms11010001 36677293 PMC9866204

[65] Shinkai T, Takizawa S, Enishi O, Higuchi K, Ohmori H, Mitsumori M. 2024. Characteristics of rumen microbiota and Prevotella isolates found in high propionate and low methane-producing dairy cows. Front Microbiol 15:1404991. doi:10.3389/fmicb.2024.1404991 38887715 PMC11180796

[66] Hernández-Vázquez A, Garcia-Arellano H, González-Cervantes RM, López-Pérez M, Soto LMH, Meza JAC, Aguirre-Garrido JF. 2026. Study of microbial communities in the soda lake of Isabel Island: identification of polyhydroxybutyrate (PHB) degrading enzymes. Environ Microbiol Rep 18:e70279. doi:10.1111/1758-2229.70279 41702408 PMC12912811

[67] Yang M, Xue Q, Zuo Z, Zhou J, Zhang S, Li M, Zhou H, Zhang M, Kumar S, Li W, et al.. 2022. Aliidiomarina halalkaliphila sp. nov., a haloalkaliphilic bacterium isolated from a soda lake in Inner Mongolia autonomous region, China. Int J Syst Evol Microbiol 72:005263. doi:10.1099/ijsem.0.005263 35244531 PMC9558577

[68] Sorokin D. Yu., Kuenen JG. 2005. Chemolithotrophic haloalkaliphiles from soda lakes. FEMS Microbiol Ecol 52:287–295. doi:10.1016/j.femsec.2005.02.012 16329914

[69] Berben T, Overmars L, Sorokin DY, Muyzer G. 2019. Diversity and distribution of sulfur oxidation-related genes in Thioalkalivibrio, a genus of chemolithoautotrophic and haloalkaliphilic sulfur-oxidizing bacteria. Front Microbiol 10:160. doi:10.3389/fmicb.2019.00160 30837958 PMC6382920

[70] Han S-B, Yu Y-H, Ju Z, Li Y, Zhang R, Hou X-J, Ma X-Y, Yu X-Y, Sun C, Wu M. 2018. Rhodohalobacter barkolensis sp. nov., isolated from a saline lake and emended description of the genus Rhodohalobacter. Int J Syst Evol Microbiol 68:1949–1954. doi:10.1099/ijsem.0.002775 29676726

[71] Soufi HH, Tran D, Louca S. 2024. Microbiology of Big Soda lake, a multi-extreme meromictic volcanic crater lake in the Nevada desert. Environ Microbiol 26:e16578. doi:10.1111/1462-2920.16578 38350645

[72] Vavourakis CD, Mehrshad M, Balkema C, van Hall R, Andrei A-Ş, Ghai R, Sorokin DY, Muyzer G. 2019. Metagenomes and metatranscriptomes shed new light on the microbial-mediated sulfur cycle in a Siberian soda lake. BMC Biol 17:69. doi:10.1186/s12915-019-0688-7 31438955 PMC6704655

[73] Sorokin DY, Detkova EN, Muyzer G. 2011. Sulfur-dependent respiration under extremely haloalkaline conditions in soda lake “acetogens” and the description of Natroniella sulfidigena sp. nov. FEMS Microbiol Lett 319:88–95. doi:10.1111/j.1574-6968.2011.02272.x 21438913

[74] Sorokin DYu, Kuenen JG. 2005. Haloalkaliphilic sulfur-oxidizing bacteria in soda lakes. FEMS Microbiol Rev 29:685–702. doi:10.1016/j.femsre.2004.10.005 16102598

[75] Xu J, Ma J, Lin H, Yan S, Niu H. 2026. Metagenomic and metabolomic analyses of rumen fiber digestion in Mongolian cattle fed fresh grass versus hay. Microbiol Spectr 14:e03051-25. doi:10.1128/spectrum.03051-25 41504449 PMC12889088

[76] Jumper J, Evans R, Pritzel A, Green T, Figurnov M, Ronneberger O, Tunyasuvunakool K, Bates R, Žídek A, Potapenko A, et al.. 2021. Highly accurate protein structure prediction with AlphaFold. Nature 596:583–589. doi:10.1038/s41586-021-03819-2 34265844 PMC8371605

[77] Zheng J, Liu H, Qin X, Yang K, Tian J, Wang X, Wang Y, Wang Y, Yao B, Luo H, et al.. 2022. Identification and mutation analysis of nonconserved residues on the TIM-barrel surface of GH5_5 cellulases for catalytic efficiency and stability improvement. Appl Environ Microbiol 88:e01046–22. doi:10.1128/aem.01046-22 36000858 PMC9469711

[78] Liew KJ, Liang CH, Lau YT, Yaakop AS, Chan K-G, Shahar S, Shamsir MS, Goh KM. 2022. Thermophiles and carbohydrate-active enzymes (CAZymes) in biofilm microbial consortia that decompose lignocellulosic plant litters at high temperatures. Sci Rep 12:2850. doi:10.1038/s41598-022-06943-9 35181739 PMC8857248

[79] Kumar R, Henrissat B, Coutinho PM. 2019. Intrinsic dynamic behavior of enzyme:substrate complexes govern the catalytic action of β-galactosidases across clan GH-A. Sci Rep 9:10346. doi:10.1038/s41598-019-46589-8 31316086 PMC6637243

[80] Macdonald SS, Blaukopf M, Withers SG. 2015. N-acetylglucosaminidases from CAZy family GH3 are really glycoside phosphorylases, thereby explaining their use of histidine as an acid/base catalyst in place of glutamic acid. J Biol Chem 290:4887–4895. doi:10.1074/jbc.M114.621110 25533455 PMC4335228

[81] Kundu S, Sharma R. 2016. In silico identification and taxonomic distribution of plant class C GH9 endoglucanases. Front Plant Sci 7. doi:10.3389/fpls.2016.01185 PMC4981690 27570528

[82] Bruno Baron C, Mon ML, Marrero Díaz de Villegas R, Cattaneo A, Di Donato P, Poli A, Negri ME, Alegre M, Soria MA, Rojo MC, et al.. 2025. Characterization of two GH10 enzymes with ability to hydrolyze pretreated Sorghum bicolor bagasse. Appl Microbiol Biotechnol 109:104. doi:10.1007/s00253-025-13484-4 40295346 PMC12037437

[83] Tu T, Li Y, Su X, Meng K, Ma R, Wang Y, Yao B, Lin Z, Luo H. 2016. Probing the role of cation-π interaction in the thermotolerance and catalytic performance of endo-polygalacturonases. Sci Rep 6:38413. doi:10.1038/srep38413 27929074 PMC5143973

[84] Mirete S, Morgante V, González-Pastor JE. 2016. Functional metagenomics of extreme environments. Curr Opin Biotechnol 38:143–149. doi:10.1016/j.copbio.2016.01.017 26901403

[85] Mesbah NM. 2022. Industrial biotechnology based on enzymes from extreme environments. Front Bioeng Biotechnol 10:870083. doi:10.3389/fbioe.2022.870083 35480975 PMC9036996

