# Supplementary figures and images for: CAZyme fold architecture is conserved between disparate environments despite extreme sequence divergence
Source: mSystems. 2026 May 19;11(6):e00485-26. doi: 10.1128/msystems.00485-26 (PMC13288935; doi:10.1128/msystems.00485-26)

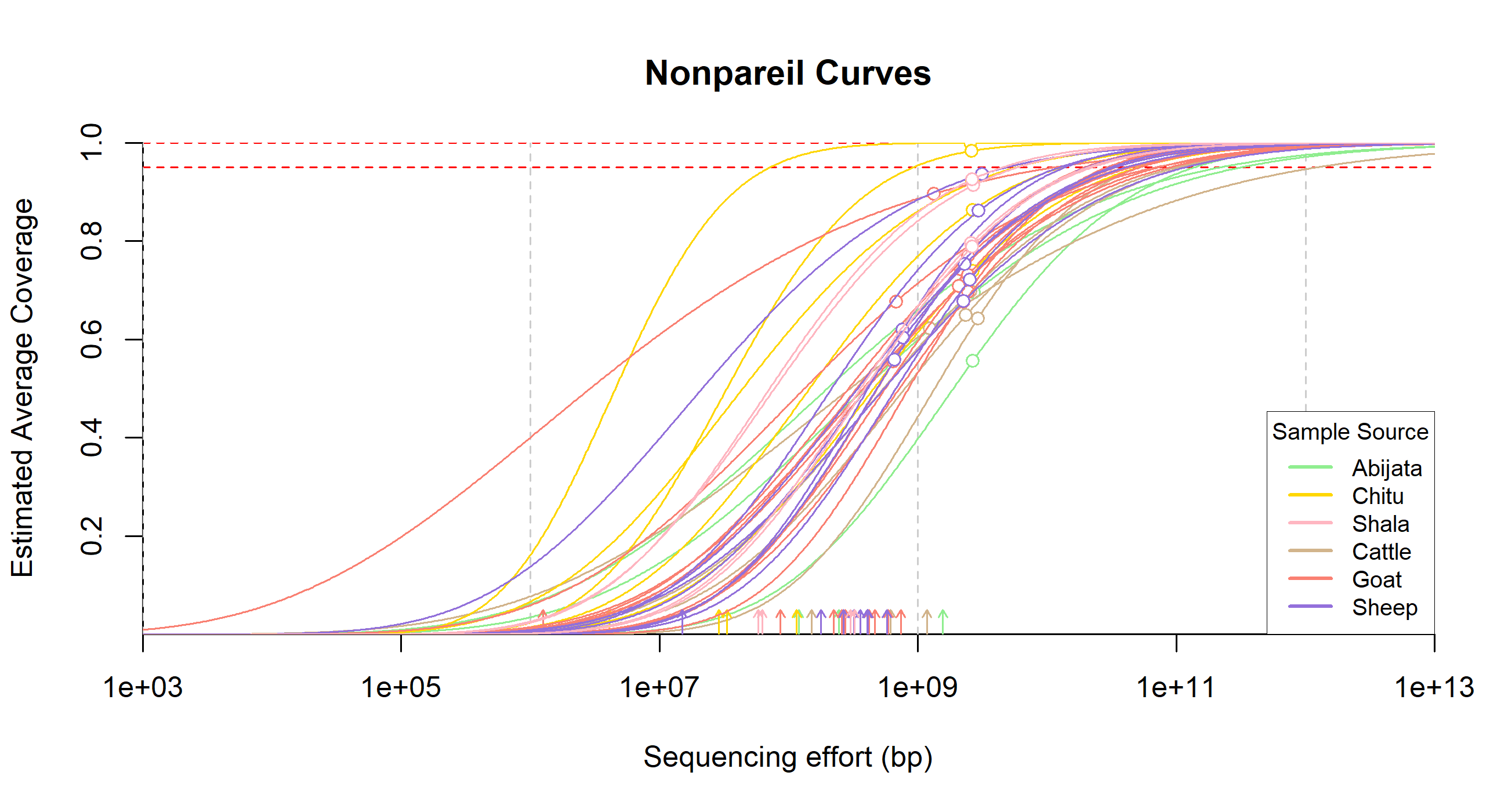

Supplement: Figure S1 — Assessment of metagenomic sequencing coverage using Nonpareil curves. [file msystems.00485-26-s0001.png]
