# Supplementary material for: CAZyme fold architecture is conserved between disparate environments despite extreme sequence divergence
Source: mSystems. 2026 May 19;11(6):e00485-26. doi: 10.1128/msystems.00485-26 (PMC13288935; doi:10.1128/msystems.00485-26)
Supplement: Legend — Supplemental figure legend. [file msystems.00485-26-s0002.docx]

**Supplementary Figure Legends**

**Supplementary Figure 1.** Assessment of metagenomic sequencing coverage using Nonpareil curves. The plot displays the estimated average coverage (y-axis) as a function of sequencing effort in base pairs (x-axis). Curves are colored by sample source (Soda Lakes: Abijata, Chitu, Shala; Rumen: Cattle, Goat, Sheep). Open circles represent the actual sequencing depth achieved for each sample. The horizontal red dashed lines indicate 95% and near 100% coverage thresholds. The plateauing of the majority of curves near or above the 95% threshold indicates that the sequencing depth was sufficient to recover the dominant microbial diversity in these communities.
